# Incidence of Total Knee Arthroplasty in Older Females with Knee Osteoarthritis and Osteoporosis Treated with Denosumab Compared with Those Treated Using Bisphosphonates: A Population-Based Cohort Study

**DOI:** 10.3390/life14121704

**Published:** 2024-12-23

**Authors:** Tzai-Chiu Yu, Wen-Tien Wu, Ru-Ping Lee, Ing-Ho Chen, Jen-Hung Wang, Shu-Hui Wen, Kuang-Ting Yeh

**Affiliations:** 1 Department of Orthopedics, Hualien Tzu Chi Hospital, Buddhist Tzu Chi Medical Foundation, Hualien 970473, Taiwan; feyu@tzuchi.com.tw (T.-C.Y.); timwu@tzuchi.com.tw (W.-T.W.); ihchen@tzuchi.com.tw (I.-H.C.); 2 Institute of Medical Sciences, Tzu Chi University, Hualien 970374, Taiwan; fish@gms.tcu.edu.tw; 3 School of Medicine, Tzu Chi University, Hualien 970374, Taiwan; 4 Department of Medical Research, Hualien Tzu Chi Hospital, Buddhist Tzu Chi Medical Foundation, Hualien 970473, Taiwan; paulwang@tzuchi.com.tw; 5 Department of Public Health, Tzu Chi University, Hualien 970374, Taiwan; 6 Graduate Institute of Clinical Pharmacy, Tzu Chi University, Hualien 970374, Taiwan

**Keywords:** bisphosphonates, older patients, denosumab

## Abstract

This study aimed to evaluate the incidence of total knee arthroplasty (TKA), a marker of severe knee osteoarthritis (OA), among older females with concurrent knee OA and osteoporosis (OP) who were treated with denosumab or bisphosphonates. By analyzing a large population-based cohort, we sought to clarify how these treatments influence the progression of knee OA to the point of requiring surgical intervention. We used data from the Taiwan National Health Insurance Research Database, including data from females aged ≥ 50 years diagnosed with knee OA and OP who initiated treatment between 2012 and 2019. Propensity score matching (1:1) resulted in the selection of 13,774 patients (6897 per group). The TKA incidence was analyzed using Cox proportional hazards models. Patients treated with denosumab had a lower TKA incidence than those treated with bisphosphonates (6.9 vs. 8.5 per 1000 person-years). The adjusted hazard ratio (aHR) for TKA in the denosumab group was 0.77 (95% CI: 0.62–0.97; *p* = 0.024), with the most pronounced effect observed in patients aged ≥ 80 years (aHR = 0.39, 95% CI: 0.20–0.77; *p* = 0.007). These findings suggest that denosumab reduces TKA risk more effectively than bisphosphonates and may serve as a superior treatment option for mitigating severe knee OA progression, especially in older adults.

## 1. Introduction

Knee osteoarthritis (OA) is a prevalent degenerative joint disease, affecting approximately 41.1% of individuals aged 60 years or older in certain populations, with its incidence and prevalence increasing significantly with age. It causes substantial public health challenges globally [1,2]. The prevalence of knee OA increases with age, with studies showing that it affects up to 10% of men and 13% of women aged 60 years or older in the United States, and these rates are expected to rise with the aging global population [3]. The prevalence of knee OA increases with age and is especially common among postmenopausal women. This is primarily due to hormonal changes that negatively impact joint health [4]. These same hormonal changes also contribute to the onset of osteoporosis, another major health concern in the older population, particularly among postmenopausal women [5]. Osteoporosis is characterized by decreased bone density and an increased risk of fractures, sharing multiple risk factors with OA, such as aging, hormonal changes, and reduced physical activity [6]. The concurrent occurrence of these two conditions in postmenopausal women presents unique challenges for treatment and patient management [7]. As knee OA progresses, joint deterioration often necessitates surgical intervention when conservative treatments, such as pain medication, physical therapy, and intra-articular injections, fail to provide adequate relief [8]. Total knee arthroplasty (TKA) is the standard surgical procedure for end-stage knee OA, offering both pain relief and functional improvement [9]. TKA also serves as a clinical indicator of severe and aggravated knee OA, representing the point at which other treatment modalities become ineffective [10]. In the context of an aging society, understanding the factors that influence the need for TKA in patients with knee OA is particularly critical, especially when comorbid conditions such as osteoporosis further complicate management strategies [11]. The poor bone quality associated with osteoporosis can adversely affect TKA outcomes and complicate treatment decisions, especially when selecting appropriate pharmacological therapies [12]. Anti-osteoporotic treatments, such as bisphosphonates and denosumab, are widely used to prevent fractures and improve bone density [13]. Bisphosphonates, including medications such as alendronate and zoledronate, inhibit bone resorption, thereby strengthening bone [14]. Denosumab, a monoclonal antibody targeting the receptor activator of nuclear factor kappa-B ligand (RANKL), also inhibits osteoclast activity and reduces bone loss, demonstrating efficacy in reducing fracture risk in postmenopausal women [15].

Although bisphosphonates and denosumab are effective in treating osteoporosis, their effect on knee OA progression and the subsequent need for TKA remain unclear. Some studies have suggested that bisphosphonates may slow OA progression by reducing subchondral bone resorption, potentially delaying the need for TKA [7,16]. However, other studies have yielded mixed results, with some reporting that there is no significant effect on OA progression [17]. Previous studies have also indicated that long-term bisphosphonate use may be associated with reduced risks of implant revision, bone loss, and adverse outcomes after joint arthroplasty [18,19,20]. Despite these findings, the effects of bisphosphonates on slowing knee OA progression and the effects of denosumab on joint health and the incidence of TKA remain underexplored. This knowledge gap leaves uncertainty regarding how these treatments influence knee OA progression in patients with coexisting osteoporosis.

Given the high prevalence of knee OA and osteoporosis in older females and the widespread use of bisphosphonates and denosumab, it is crucial to investigate how these treatments affect knee OA progression to the point of requiring TKA. We propose that the incidence of TKA can serve as a marker of severe and aggravated knee OA. Investigating whether osteoporosis treatments modify the risk of TKA could provide valuable insights into the long-term management of these patients and inform clinical decision-making regarding anti-osteoporotic therapy choices.

Therefore, this study aimed to evaluate the incidence of TKA, representing severe knee OA, among older females diagnosed with knee OA and osteoporosis who were receiving treatment with either denosumab or bisphosphonates. We hypothesized that there may be differences in the TKA risk between the two treatment groups when considering their different mechanisms of action on bone resorption and joint health. By analyzing data from the Taiwan National Health Insurance Research Database (NHIRD), we aimed to clarify the relationship between osteoporosis treatment and the need for TKA, which can serve as an indicator of aggravated knee OA.

## 2. Materials and Methods

### 2.1. Ethics Statements

Ethical approval was obtained for this study from the Research Ethics Committee of the Hualien Tzu Chi Hospital (institutional review board number: 108-242-C). The need for informed consent was waived by the Research Ethics Committee because the big data used here were anonymized.

### 2.2. Study Design and Population

This study employed a retrospective cohort design, drawing data from the Taiwan NHIRD managed by the National Health Insurance Administration, Ministry of Health and Welfare. The NHIRD provides extensive medical information on >99% of Taiwan’s population, making it an invaluable resource for large-scale population-based research [21]. This dataset includes comprehensive information on patient demographics, diagnostics, treatments, and medical procedures. Data were extracted from patients diagnosed with knee OA and osteoporosis between 1 January 2012 and 31 December 2019.

**Figure 1:**
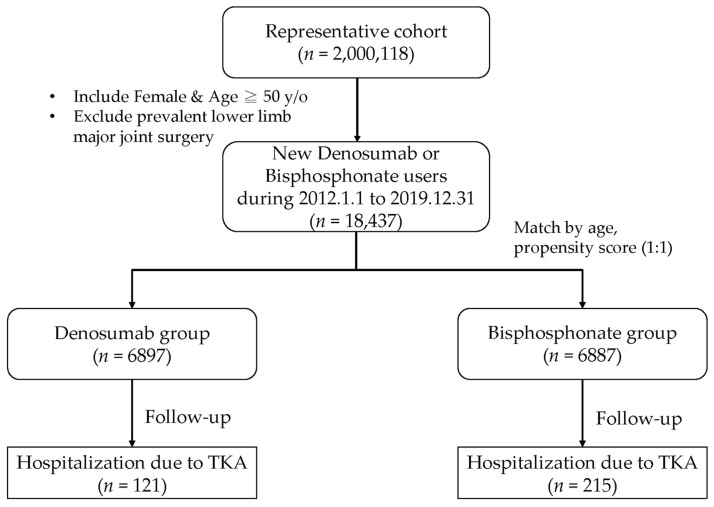
The flowchart of this cohort study.

This study included females aged ≥ 50 years who had been diagnosed with knee OA and osteoporosis and had initiated anti-osteoporotic treatment during the study period. Specifically, the cohort comprised patients who began treatment with either denosumab or any type of bisphosphonate (e.g., alendronate, risedronate, and zoledronate). To ensure data validity, we excluded patients with a history of major lower-limb joint surgery, including TKA, before their osteoporosis diagnosis or prior to their index date of treatment initiation. Additionally, individuals aged < 50 years were excluded because knee OA and osteoporosis predominantly affect the older population. The initial cohort was drawn from 2,000,118 individuals from the general population of the NHIRD. Among these, 18,437 were new users of denosumab or bisphosphonates who began use during the study period. The denosumab group included 6897 patients, whereas the bisphosphonate group consisted of 6887 patients. To ensure comparability, the groups were matched for age, sex, and propensity score at a 1:1 ratio (Figure 1).

### 2.3. Outcomes and Data Collection

The primary outcome was the incidence of TKA, which served as an indicator of severe and aggravated knee OA requiring surgical intervention. TKA was defined as hospitalization due to knee replacement surgery after the initiation of anti-osteoporotic treatment. As TKA marks a stage where conservative management is insufficient, it is considered a reliable and objective marker of knee OA progression that necessitates surgical intervention.

### 2.4. Statistical Analysis

To ensure balanced characteristics between the denosumab and bisphosphonate groups, the patients were matched for age and the index year of treatment initiation. Propensity score matching was also applied to adjust for baseline characteristics that could influence the relationship between the treatment type and TKA risk. Propensity scores were calculated on the basis of factors such as age and relevant comorbidities (e.g., hypertension, diabetes mellitus, coronary artery disease, and cerebrovascular disease). Individuals with a history of major lower-limb joint surgery, considerable trauma, or fractures unrelated to osteoporosis were excluded. Patients with incomplete records or patients lost to follow-up were excluded from the final analysis. We assessed the balance of baseline characteristics between the denosumab and bisphosphonate groups after propensity score matching by calculating the standardized mean difference (SMD) for each variable. An SMD of less than 0.1 was considered indicative of a negligible difference between the groups, ensuring that the matching process was effective in minimizing potential confounding factors. To ensure that the statistical power is adequate for detecting significant differences in the incidence of TKA between the groups, we performed a sample size calculation for survival analysis. Assuming an α level of 0.05, β of 0.2, a hazard ratio of 0.76, and an equal proportion of subjects in both groups, we determined that at least 417 total events were required. Based on a median survival time of 10 years in the bisphosphonate group, a censoring rate of 0.31, and an average planned follow-up duration of 3.13 years, the minimum total sample size required was estimated to be 3697.

Continuous variables are summarized as means and standard deviations, whereas categorical variables are presented as counts and percentages. For comparisons between groups, continuous variables were analyzed using the Student’s *t*-test, whereas categorical variables were assessed using the chi-square or Fisher exact test, as appropriate. The incidence of TKA was calculated for the denosumab and bisphosphonate groups and expressed as TKA cases per 1000 person-years. Cox proportional hazard models were used to estimate the crude and adjusted hazard ratios (aHRs) for TKA risk, with bisphosphonates serving as the reference group. The aHRs were derived using multivariate models that controlled for baseline characteristics, e.g., age, comorbidities, and follow-up duration. To account for potential competing risks, such as death, a competing risk analysis was conducted to ensure a robust outcome assessment. All statistical analyses were performed using SAS software (version 9.4; SAS Institute, Cary, NC, USA) and Stata (version 16; StataCorp, College Station, TX, USA). Statistical significance was determined at a *p*-value < 0.05.

## 3. Results

### 3.1. Study Population

This study included 13,774 patients, with 6897 in the denosumab group and 6887 in the bisphosphonate group, matched for age and propensity score. The demographic and clinical characteristics of the two groups are shown in Table 1. The SMDs for all variables were below 0.1, indicating effective matching and minimal baseline differences between the groups. The power analysis confirmed that the study’s sample size was adequate to detect statistically significant differences in TKA risk between the treatment groups. With a total of 13,774 patients and 336 TKA events, the study far exceeded the calculated minimum required sample size of 3697 participants, ensuring robust statistical power. The mean age of the study population was 74.34 ± 9.44 years, with no significant difference between the groups (*p* = 0.076). The distribution of patients by age group was also similar, with 50.6% of patients in the 65–80-year-old age group and 32.8% in the ≥80-year-old group. Comorbidities such as hypertension, diabetes mellitus, hyperlipidemia, and coronary artery disease were comparable between the two groups, with no significant differences observed in the prevalence of these conditions (all, *p* > 0.05). The denosumab and bisphosphonate groups had similar follow-up periods (2.58 ± 1.95 years and 3.74 ± 2.36 years, respectively; *p* < 0.001) (Table 1).

### 3.2. Incidence of TKA

A total of 336 patients underwent TKA during the follow-up period: there were 121 (1.8%) in the denosumab group and 215 (3.1%) in the bisphosphonate group (Table 1). The incidences of TKA were 6.9 and 8.5 per 1000 person-years in the denosumab and bisphosphonate groups, respectively (Table 2).

### 3.3. Risk of TKA

**Figure 2:**
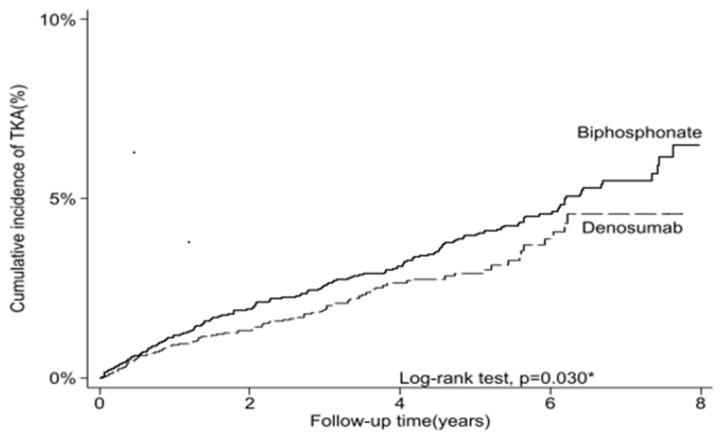
The cumulative incidence of total knee arthroplasty of female patients with knee osteoarthritis and osteoporosis who have received bisphosphonates or denosumab treatment (*n* = 13,774). * *p*-value < 0.05 was considered statistically significant after test.

In the univariate analysis, the crude hazard ratio (HR) for TKA was 0.78 in the denosumab group compared to the bisphosphonate group (95% CI: 0.62–0.98; *p* = 0.030) (Table 2). After adjusting for baseline characteristics using multivariate Cox proportional hazards models, the aHR was 0.77 (95% CI: 0.62–0.97; *p* = 0.024) (Table 2). A competing risk analysis, which accounted for the possibility of non-TKA-related events, such as death, also revealed that there was a significantly lower risk of TKA in the denosumab group than in the bisphosphonate group (aHR = 0.76, 95% CI: 0.61–0.96; *p* = 0.020) (Table 2). The cumulative incidence of TKA was compared between patients receiving bisphosphonates and those receiving denosumab over an 8-year follow-up period (Figure 2). The analysis revealed that there was a statistically significant difference between the two groups (log-rank test, *p* = 0.030). Patients treated with bisphosphonates demonstrated a higher cumulative incidence of TKA, reaching approximately 7% by the end of the 8-year follow-up period, whereas the denosumab group showed a relatively lower rate, stabilizing at approximately 4.5%. Both groups exhibited a steeper increase in the incidence of TKA during the first 2 years of follow-up, followed by a more gradual increase thereafter. The divergence between the two treatment groups became more apparent after the fourth year of follow-up, with the bisphosphonate group consistently showing a higher cumulative incidence of TKA than the denosumab group (Figure 2).

### 3.4. Subgroup Analysis by Age

Subgroup analysis was conducted to examine the risk of TKA in different age groups. For patients aged 50–65 years, there was no statistically significant difference in the risk of TKA between the denosumab and bisphosphonate groups, with an aHR of 1.49 (95% CI: 0.78–2.86; *p* = 0.228) (Table 3). In the 65–80-year-old group, denosumab treatment was associated with a significantly lower risk of TKA than bisphosphonate treatment, with an aHR of 0.77 (95% CI: 0.59–0.99; *p* = 0.045). The most pronounced effect was observed in the ≥80-year-old group, where denosumab was associated with a substantial reduction in TKA risk (adjusted HR = 0.39, 95% CI: 0.20–0.77; *p* = 0.007) (Table 3).

## 4. Discussion

In this study, patients treated with denosumab had a significantly lower risk of TKA than those treated with bisphosphonates, suggesting the possible protective effect of denosumab on knee joint health in comparison with bisphosphonates. This was particularly evident in the subgroup of patients aged ≥ 80 years, where denosumab was associated with a substantial reduction in TKA risk. These findings align with the growing interest in the differential effects of osteoporosis treatments on joint health, especially in individuals with concurrent musculoskeletal conditions, e.g., knee OA [14].

### 4.1. Denosumab and Reduced Incidence of TKA

The primary study finding was the association of denosumab with a lower incidence of TKA compared with bisphosphonates. The aHR for TKA in the denosumab group was 0.77, indicating a 23% reduction in the risk of severe knee OA requiring surgical intervention compared with the bisphosphonate group. These results support the hypothesis that denosumab, through the inhibition of RANKL and the suppression of osteoclast activity, may have beneficial effects on subchondral bone health, potentially slowing OA progression and delaying TKA [17]. The RANK/RANKL/OPG signaling pathway plays a crucial role in OA pathogenesis, where an imbalance leads to excessive osteoclast activity, resulting in bone resorption and cartilage degradation. By inhibiting RANKL, denosumab reduces osteoclast-mediated bone resorption and potentially alleviates the structural damage typical of OA [22,23]. Studies have demonstrated the efficacy of denosumab in the clinical setting, particularly in reducing the structural progression of erosive hand OA. In a randomized placebo-controlled trial, denosumab significantly prevented the development of new erosive joints and promoted subchondral bone remodeling, highlighting its role in the structural preservation of OA-affected joints [24]. Further experimental studies have provided insights into how denosumab mitigates OA progression by protecting the cartilage through the suppression of osteoclastogenesis and a reduction in chondrocyte apoptosis. These cellular processes are critical contributors to cartilage degradation and subchondral bone remodeling in OA. By modulating these processes, denosumab helps to preserve the integrity of the articular cartilage and subchondral bone, thereby delaying OA progression and potentially reducing the need for surgical interventions, such as TKA [17]. In summary, denosumab is a viable therapeutic agent for older females with OA, particularly those with osteoporosis. Its dual role in managing bone density and protecting joint structures underscores its potential to alter the OA disease trajectory, offering considerable advantages in the management of degenerative joint disease [16].

### 4.2. Comparative Effect of Bisphosphonates on the TKA Risk

In contrast to denosumab, bisphosphonates did not show a significant protective effect against knee OA progression to the point where TKA was required. The incidence rate of TKA in the bisphosphonate group was 8.5 per 1000 person-years compared with 6.9 per 1000 person-years in the denosumab group. These results align with the mixed findings in the literature regarding the effects of bisphosphonates on OA progression. While some studies over the past decade have indicated that bisphosphonates may be beneficial for patients undergoing TKA or total hip replacement by reducing perioperative complications and implant loosening [18,19,20,25,26,27], their role in slowing OA progression remains contentious. Preclinical studies have suggested that bisphosphonates, e.g., alendronate, may slow or reverse OA progression by stabilizing subchondral bone, reducing microdamage, and suppressing inflammatory cytokines that contribute to cartilage degradation [28,29]. However, clinical evidence has shown inconsistent outcomes. A 4-year study for the Osteoarthritis Initiative showed that there was no significant association between bisphosphonate use and the prevention or delay of radiographic changes and pain in hip OA, highlighting the variability in response among different OA types and the limitations of bisphosphonates in managing structural OA progression [30]. The effects of bisphosphonates on cartilage and subchondral bone structures appear to be context-dependent. Although some studies have demonstrated reduced osteophyte formation and cartilage loss in animal models, others have demonstrated limited efficacy in human clinical settings [31]. The complexity of OA pathophysiology, involving changes in the bone and cartilage, suggests that bisphosphonates may benefit specific OA phenotypes characterized by high bone turnover. For instance, bisphosphonates have shown potential in managing subchondral bone sclerosis and reducing associated cartilage degradation, which are crucial aspects of OA progression [32]. Yet, these benefits are not universally observed, and their clinical relevance in broader OA populations remains uncertain. Additionally, a review of osteoporosis treatments, including bisphosphonates, indicated promise in mitigating OA symptoms and progression, but emphasized the need for more targeted research to draw definitive conclusions [16]. This underscores the need for patient-specific treatment strategies and the potential role of bisphosphonates in comprehensive OA management plans, particularly for patients with concurrent osteoporosis.

### 4.3. Age-Dependent Effects of Denosumab Versus Bisphosphonates on TKA Risk

Our subgroup analysis revealed age-related differences in the TKA risk between the denosumab and bisphosphonate groups. In patients aged 50–65 years, there was no significant difference in the TKA risk between the treatments. However, in the 65–80-year-old group, denosumab was associated with a significant reduction in TKA risk compared with bisphosphonates. The most pronounced effect was observed in patients aged ≥ 80 years, where denosumab was associated with a 61% reduction in TKA risk. This age-related trend may reflect the cumulative impact on long-term bone health and joint integrity in older adults, where the potent antiresorptive effects of denosumab are more beneficial in slowing OA progression [17]. The significant effect of denosumab in the oldest age group may be attributed to several factors. First, older patients with osteoporosis are at greater risk of bone fragility, which exacerbates knee OA effects by contributing to subchondral bone deterioration and joint instability. The ability of denosumab to preserve bone density and inhibit bone resorption may mitigate these effects and reduce the need for surgical intervention. Additionally, older patients may have more advanced OA at baseline, where the potent suppression of bone turnover caused by denosumab may be more effective in slowing further joint degeneration than bisphosphonates [33]. These findings suggest that denosumab may be particularly beneficial in older patients with concurrent knee OA and osteoporosis, a population at a high risk of joint- and bone-related complications.

### 4.4. Influence of Sociodemographic Factors on Study Outcomes

The recent literature has highlighted the influence of sociodemographic factors, such as income, education, and urban versus rural residency, on health outcomes and treatment effectiveness [34]. For example, lower socioeconomic status is associated with reduced access to healthcare, lower adherence to prescribed treatments, and greater delays in seeking medical attention, all of which could affect the progression of conditions like knee OA and the need for surgical intervention [35,36]. Similarly, disparities in education and health literacy can impact patients’ understanding of treatment regimens, potentially influencing outcomes [37]. Although our study was unable to include these sociodemographic factors due to limitations in the Taiwan NHIRD dataset, the robustness of our propensity score matching process, which accounted for baseline comorbidities and other relevant clinical characteristics, enhances the validity of our results. Moreover, the large sample size and significant differences observed between the denosumab and bisphosphonate groups provide meaningful insights into the progression of knee OA in this population. While residual confounding by unmeasured sociodemographic variables cannot be completely ruled out, the consistency of our findings with prior studies on the effectiveness of these treatments suggests that our conclusions remain valid and relevant. Future research incorporating datasets with comprehensive sociodemographic information could further elucidate the interplay between these factors and treatment outcomes, thereby building upon the foundation established by our study.

### 4.5. Strengths and Limitations

The primary strength of this study lies in its large population-based cohort derived from the Taiwan NHIRD. The use of a nationwide database enabled robust analysis with a substantial sample size, enhancing the generalizability of our findings across diverse patient populations. The matched design and propensity score adjustment further minimized potential confounding factors, ensuring that there was a balanced comparison between the denosumab and bisphosphonate groups.

However, several limitations must be acknowledged. First, the NHIRD database does not include detailed types of sociodemographic information, such as income level, education, or urban/rural residency, which can act as potential confounders. These factors are known to influence access to healthcare, treatment adherence, and overall health outcomes. The lack of this information limited our ability to adjust for these variables, and it is possible that unmeasured sociodemographic factors contributed to any residual confounding present in our results. Future studies incorporating datasets with comprehensive sociodemographic data could provide additional insights into how these variables interact with treatment effectiveness and knee osteoarthritis progression. Second, the NHIRD also lacks certain clinical details, such as body mass index, bone mineral density, and baseline knee OA severity, which are critical factors influencing disease progression and treatment outcomes. Although propensity score matching and multivariate analyses adjust for available baseline characteristics, the absence of these variables may limit the interpretation of our findings. Third, the retrospective nature of the study inherently restricts the ability to establish causality between treatment and TKA risk. While robust statistical adjustments were employed, including the use of competing risk models, the potential for unmeasured confounders remained. Finally, the study did not include an untreated control group of OA patients, as the focus was specifically on evaluating the relative effectiveness of denosumab and bisphosphonates. Including such a control group could have provided additional context regarding the natural progression of knee OA without pharmacological intervention. This limitation is noted, and future studies incorporating untreated controls are recommended to better contextualize the benefits of these treatments.

Overall, despite these limitations, the large sample size, robust statistical adjustments, and nationwide database used in this study provide valuable insights into the effects of denosumab and bisphosphonates on TKA risk in patients with knee OA and osteoporosis. Addressing these limitations in future research will help to strengthen the evidence base and refine treatment strategies.

### 4.6. Clinical Implications and Future Research

Our findings have significant clinical implications for the management of patients with knee OA and osteoporosis. Denosumab appears to offer substantial benefits, not only in reducing fracture risk but also in lowering the incidence of TKA. These results suggest that denosumab may be a preferable treatment option for patients with concurrent OA and osteoporosis, particularly among older populations who are at higher risk of joint deterioration and display poor bone quality. The subgroup analysis by age revealed a notable trend, with the greatest protective effect of denosumab observed in patients aged 80 years and older. This finding underscores the importance of age as a critical factor in OA progression and treatment outcomes. In older individuals, who often present with more advanced joint degeneration and compromised bone health, the potent anti-resorptive effects of denosumab may provide enhanced protection against severe OA progression, delaying the need for surgical intervention. In contrast, no significant differences between the treatment groups were observed in younger patients aged 50–65 years, which was likely due to their relatively better baseline joint and bone health. These insights emphasize the need for personalized treatment strategies tailored to the patient’s age and disease severity. Clinicians should consider the dual benefits of denosumab in modulating bone and joint health when selecting anti-osteoporotic treatments, particularly for older patients with advanced OA.

Future research should focus on prospective, longitudinal studies to validate the benefits of denosumab observed across different age groups. Investigations into the underlying mechanisms of its effects on subchondral bone remodeling and cartilage preservation will further enhance our understanding of its role in OA management. Additionally, studies that integrate sociodemographic factors, untreated control groups, and longer follow-up durations will provide deeper insights into the broader applicability of these findings and help to refine treatment strategies for patients with and without osteoporosis.

## 5. Conclusions

In this large population-based cohort study, we found that denosumab was associated with a significantly lower incidence of TKA compared to bisphosphonates in older females with knee OA and osteoporosis. The adjusted hazard ratio (aHR = 0.77, 95% CI: 0.62–0.97; *p* = 0.024) indicates that denosumab reduces the risk of TKA by 23% compared to bisphosphonates, highlighting its protective effect in delaying severe knee OA progression that requires surgical intervention. These findings suggest that the observed differences in TKA incidence are driven by the superior efficacy of denosumab in mitigating joint deterioration and preserving subchondral bone health, rather than by bisphosphonates increasing the risk of TKA. The results underscore the potential of denosumab as a dual-purpose treatment for patients with coexisting OA and osteoporosis, particularly in older populations who are at high risk of joint- and bone-related complications. Future research is needed to further elucidate the mechanisms underlying the protective effects of denosumab on joint health and to explore its role in OA patients without osteoporosis. Additionally, prospective studies with longer follow-up periods and the consideration of untreated control groups will help to validate these findings and refine treatment strategies for this population.

## Figures and Tables

**Table 1:** Demographics (*n* = 13,774).

Females with OA and OP	Denosumab	Bisphosphonate	Total	*p*-Value	SMD
N	6887	6887	13774		
Age	74.49 ± 9.43	74.20 ± 9.44	74.34 ± 9.44	0.076	−0.03
Age group	-	-	-	0.884	0.02
50–65 y/o	1153 (16.7%)	1140 (16.5%)	2293 (16.6%)		
65–80 y/o	3490 (50.7%)	3477 (50.5%)	6967 (50.6%)		
≥80 y/o	2244 (32.6%)	2270 (33.0%)	4514 (32.8%)		
HTN (%)	3466 (50.3%)	3505 (50.9%)	6971 (50.6%)	0.506	0.01
DM (%)	1561 (22.7%)	1516 (22.0%)	3077 (22.3%)	0.357	−0.02
Hyperlipidemia (%)	1785 (25.9%)	1744 (25.3%)	3529 (25.6%)	0.424	−0.01
CAD (%)	847 (12.3%)	825 (12.0%)	1672 (12.1%)	0.566	−0.01
CVA (%)	768 (11.2%)	766 (11.1%)	1534 (11.1%)	0.957	0.00
Chronic liver disease (%)	380 (5.5%)	358 (5.2%)	738 (5.4%)	0.405	−0.01
Chronic renal failure (%)	0 (0.0%)	0 (0.0%)	0 (0.0%)	1.000	0.00
Depression (%)	726 (10.5%)	725 (10.5%)	1451 (10.5%)	0.978	0.00
RA (%)	183 (2.7%)	163 (2.4%)	346 (2.5%)	0.276	−0.02
TKA (%)	121 (1.8%)	215 (3.1%)	336 (2.4%)	<0.001 *	0.09
Follow-up time (year)	2.58 ± 1.95	3.74 ± 2.36	3.16 ± 2.24	<0.001 *	0.53

Data are presented as *n* or mean ± standard deviation. * *p*-value < 0.05 was considered statistically significant after test. Abbreviations: OA, knee osteoarthritis; OP, osteoporosis.

**Table 2:** Risk of TKA among female patients with OA and OP (*n* = 13774).

Variables	Denosumab	Bisphosphonate
Patient numbers	6887	6887
TKA cases	121	215
Person-years	17,604	25,285
Incidence rate ^a^	6.9	8.5
Univariate model		
Crude HR (95% CI)	0.78 (0.62–0.98)	1 (ref.)
*p*-value	0.030 *	
Multivariate model ^b^		
aHR (95% CI)	0.77 (0.62–0.97)	1 (ref.)
*p*-value	0.024 *	
Competing risk ^b^		
aHR (95% CI)	0.76 (0.61–0.96)	1 (ref.)
*p*-value	0.020 *	

^a^ per 1000 person-years. ^b^ multivariate Cox proportional hazard regression model with adjustment for all baseline characteristics shown in Table 1. * *p*-value < 0.05 was considered statistically significant after test. Abbreviations: TKA, total knee arthroplasty; OA, knee osteoarthritis; OP, osteoporosis; HR, hazard ratio; CI, confidence interval; aHR, adjusted hazard ratio; ref, reference.

**Table 3:** Subgroup analysis of different age groups (*n* = 13774).

Variables	Crude HR ^a^ (95% CI)	*p*-Value	Adjusted HR ^a^ (95% CI)	*p*-Value
Main model				
No	1.00		1.00	
Yes	0.78 (0.62–0.98)	0.030 *	0.77 (0.62–0.97)	0.024 *
Age				
50–65 y/o				
No	1.00		1.00	
Yes	1.58 (0.83–3.02)	0.164	1.49 (0.78–2.86)	0.228
65–80 y/o				
No	1.00		1.00	
Yes	0.77 (0.60–1.01)	0.055	0.77 (0.59–0.99)	0.045 *
≥80 y/o				
No	1.00		1.00	
Yes	0.39 (0.20–0.78)	0.007 *	0.39 (0.20–0.77)	0.007 *

^a^ Cox’s proportional hazards model. Abbreviations: HR, hazard ratio; CI, confidence interval. * *p*-value < 0.05 was considered statistically significant after test.

## Data Availability

All generated data were within this article.
